# The Importance of Biologically Active Vitamin D for Mineralization by Osteocytes After Parathyroidectomy for Renal Hyperparathyroidism

**DOI:** 10.1002/jbm4.10234

**Published:** 2019-10-23

**Authors:** Aiji Yajima, Ken Tsuchiya, David B Burr, Joseph M Wallace, John D Damrath, Masaaki Inaba, Yoshihiro Tominaga, Shigeru Satoh, Takashi Nakayama, Tatsuhiko Tanizawa, Hajime Ogawa, Akemi Ito, Kosaku Nitta

**Affiliations:** ^1^ Department of Anatomy and Cell Biology Indiana University School of Medicine Indianapolis IN USA; ^2^ Department of Medicine, Kidney Center Tokyo Women's Medical University Shinjuku‐ku, Tokyo Japan; ^3^ Department of Blood Purification, Kidney Center Tokyo Women's Medical University, Shinjuku‐ku Tokyo Japan; ^4^ Department of Biomedical Engineering Indiana University, Purdue University Indianapolis IN USA; ^5^ Department of Metabolism, Endocrinology and Molecular Medicine Osaka City University Graduate School of Medicine Osaka Japan; ^6^ Department of Transplant Surgery Nagoya Second Red Cross Hospital Nagoya, Aichi Japan; ^7^ Center for Kidney Disease and Transplantation Akita University Hospital Akita Japan; ^8^ Department of Orthopedic Surgery Towa Hospital Adachi‐ku, Tokyo Japan; ^9^ Department of Orthopedic Surgery Tanizawa Clinic Niigata Japan; ^10^ Department of Medicine, Division of Nephrology Ogawa Clinic Shinagawa‐ku, Tokyo Japan; ^11^ Ito Bone Histomorphometry Institute Niigata Japan

**Keywords:** MINERALIZATION, OSTEOCYTE, VITAMIN D

## Abstract

Hypomineralized matrix is a factor determining bone mineral density. Increased perilacunar hypomineralized bone area is caused by reduced mineralization by osteocytes. The importance of vitamin D in the mineralization by osteocytes was investigated in hemodialysis patients who underwent total parathyroidectomy (PTX) with immediate autotransplantation of diffuse hyperplastic parathyroid tissue. No previous reports on this subject exist. The study was conducted in 19 patients with renal hyperparathyroidism treated with PTX. In 15 patients, the serum calcium levels were maintained by subsequent administration of alfacalcidol (2.0 μg/day), i.v. calcium gluconate, and oral calcium carbonate for 4 weeks after PTX (group I). This was followed in a subset of 4 patients in group I by a reduced dose of 0.5 μg/day until 1 year following PTX; this was defined as group II. In the remaining 4 patients, who were not in group I, the serum calcium (Ca) levels were maintained without subsequent administration of alfacalcidol (group III). Transiliac bone biopsy specimens were obtained in all groups before and 3 or 4 weeks after PTX to evaluate the change of the hypomineralized bone area. In addition, patients from group II underwent a third bone biopsy 1 year following PTX. A significant decrease of perilacunar hypomineralized bone area was observed 3 or 4 weeks after PTX in all group I and II patients. The area was increased again in the group II patients 1 year following PTX. In group III patients, an increase of the hypomineralized bone area was observed 4 weeks after PTX. The maintenance of a proper dose of vitamin D is necessary for mineralization by osteocytes, which is important to increase bone mineral density after PTX for renal hyperparathyroidism. © 2019 The Authors. *JBMR Plus* published by Wiley Periodicals, Inc. on behalf of American Society for Bone and Mineral Research.

## Introduction

**Figure 1:**
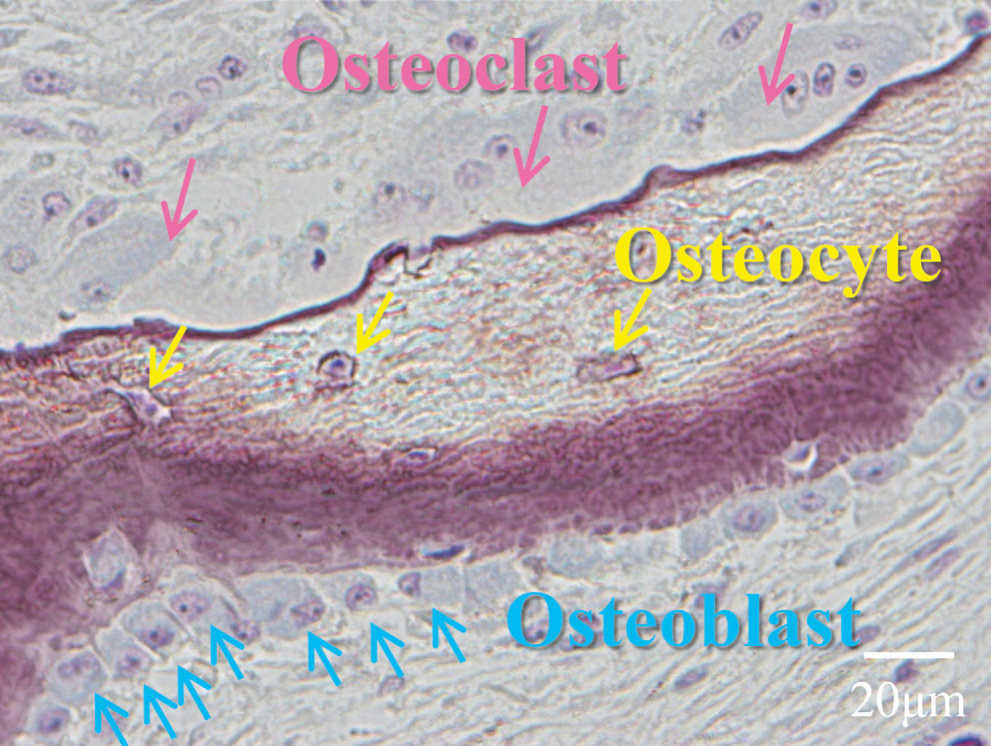
Osteoclasts, osteoblasts, and osteocytes in patients with renal hyperparathyroidism. The relationships between osteocytes and both osteoclasts and osteoblasts in patients with renal hyperparathyroidism are shown.

Accumulated evidence suggests that the osteocyte is a crucial determinant of bone strength. The osteocyte network spreads extensively over the skeleton, controlling osteocytic perilacunar/canalicular turnover and regulating some functions of osteoclasts and osteoblasts. Figure 1 shows the relationships between osteocytes and both osteoclasts and osteoblasts in HD patients with renal hyperparathyroidism.^1^, ^2^, ^3^ Nevertheless, there have been few clinical studies about the role of the osteocyte in patients with chronic kidney disease (CKD).^4^, ^5^, ^6^, ^7^ It is likely that bone fragility in CKD patients suffering from renal hyperparathyroidism increases^8^, ^9^ as a result of increased hypomineralized bone area caused by impaired mineralization by osteocytes,^10^, ^11^ increased woven bone area,^12^, ^13^ and the reduced cortical bone area caused by cortical thinning and increased cortical porosity.^14^, ^15^ The long‐term reduction of osteocyte numbers generally leads to increased fracture risk.^16^

Parathyroidectomy is an important treatment option for hyperparathyroid bone disease caused by renal hyperparathyroidism^17^, ^18^ because this procedure is completed in approximately 1 hour with little bleeding. However, whether mineralized bone area generally increases following parathyroidectomy has not been clarified. It was reported that parathyroidectomy with or without subsequent vitamin D administration increases bone mineral and reduces the fracture risk of dialysis patients,^17^, ^18^ but the importance of subsequent administration of biologically active vitamin D after parathyroidectomy on mineralization on lacunar surfaces by osteocytes has not been well‐studied. Total parathyroidectomy (PTX) with immediate autotransplantation of 150 mg of diffuse hyperplastic parathyroid tissue is really important in patients with severe renal hyperparathyroidism.^7^, ^19^ Osteoblast surface (Ob.S/BS) increases 1 week after parathyroidectomy, and low‐turnover osteomalacia with severe hypophosphatemia transiently develops 4 weeks after the surgery; thereafter, the serum phosphorus (P) level gradually increases. And BMD is significantly increased after parathyroidectomy as presented in previously published articles.^7^, ^18^, ^19^ Thus, mineralization by osteocytes is critical to increase BMD in CKD patients; its importance has been demonstrated in both CKD patients and animal models without CKD.^1^, ^2^, ^3^, ^4^, ^7^

We have demonstrated increased osteocyte death and mineralization at the osteocytic perilacunar/canalicular surface after PTX.^7^ And the degree of mineralization at the osteocytic perilacunar/canalicular surface is greater than that at the mineralization front by osteoblasts after PTX with subsequent administration of 2.0 μg/day of alfacalcidol (1α‐hydroxyvitamin D_3_; Chugai Pharma Co. Ltd., Tokyo, Japan).^7^ The existence of the parathyroid hormone receptor^20^ and the effects of biologically activated vitamin D on the vitamin D receptor^21^ expressed by the osteocyte may affect these changes. Moreover, the number of osteocytes is markedly greater than that of osteoblasts,^22^ and there is significantly more surface area for molecular exchange associated with osteocyte lacunae and canaliculi (1200 m^2^) than that found in Haversian/Volkmann's canals (3.2 m^2^) or cancellous surfaces (9.0 m^2^) in the male skeleton.^23^

The importance of activated vitamin D for mineralization by osteocytes in CKD patients must be explored. In this study, the change of hypomineralized bone area was analyzed in HD patients with and without vitamin D supplementation after PTX to ascertain the importance of vitamin D in perilacunar/canalicular mineralization by osteocytes.

## Materials and Methods

### Study design

Six women and 13 men with an average age of 58.4 ± 8.8 (39–71) years who were under maintenance by HD were enrolled in this study. They had been on HD for an average of 14.1 ± 7.3 (1 to 25) years. Because the sensitivity of bone tissue to PTH is reduced in patients with diabetes mellitus,^24^, ^25^ none of the patients in this clinical study suffered from diabetes mellitus. If the serum levels of intact PTH were >1000 pg/mL or total alkaline phosphatase was >360 U/L in the HD patients, the patients with renal hyperparathyroidism were treated with PTX. This was usually because the condition was refractory to conservative therapy with vitamin D sterols. We included all patients who received PTX between 1996 and 2010. No patients received cinacalcet hydrochloride (HCl) (Amgen Inc., Thousand Oaks, CA, USA, and Kyowa Hakko Kirin, Tokyo, Japan) treatment before PTX (Table 1).

**Table 1:** Characteristics of HD Patients in This Study

	Total (*n* = 19)	Group I (*n* = 15)	Group II (*n* = 4)	Group III (*n* = 4)
Age (years)	58.4 ± 8.8 (39–71)	57.1 ± 9.3 (39–71)	52.8 ± 5.9 (45–59)	63.3 ± 5.2 (56–67)
Duration of HD (years)	14.1 ± 7.3 (1–25)	12.9 ± 7.5 (1–25)	12.0 ± 8.8 (2–23)	18.5 ± 4.7 (15–25)
Kidney disease (*n*)	12; 1; 1; 5	11; 1; 1; 2	2; 0; 0; 2	1; 0; 0; 3
Female; male (*n*)	6; 13	6; 9	1; 3	0; 4
Bone biopsies		Pre‐PTX	Pre‐PTX	Pre‐PTX
		Post‐PTX (3.8 ± 0.4 weeks)	Post‐PTX (4 weeks)	Post‐PTX (4 weeks)
			Post‐PTX (1 year)	

Nineteen HD patients were divided into three groups: group I, II, and III. Kidney disease included chronic glomerular nephritis, polycystic kidney disease, hypertension, and unknown. All variables are expressed as mean ± SD (*n* = 19).

HD = hemodialysis; PTX = total parathyroidectomy.

The 19 patients with renal hyperparathyroidism were divided into the three groups: group I, consisted of patients (*n* = 15) who received subsequent administration of 2.0 μg/day of alfacalcidol for 3 to 4 weeks after PTX; group II, consisted of a subset of 4 patients from group I who received subsequent administration of 2.0 μg/day of alfacalcidol for 4 weeks after PTX, but a reduced dose of alfacalcidol (0.5 μg/day) from 4 weeks to 1 year following PTX; group III, consisted of a separate group of patients (*n* = 4) did not receive alfacalcidol administration postoperatively because of a vitamin D allergy or to avoid extraosseous ossification caused by high vitamin D levels after surgery.^26^ Serum PTH levels must be increased to avoid long‐term low‐turnover bone disease to avoid increased microcracks and soft tissue ossification after PTX.^27^, ^28^ Ca‐containing drugs, including i.v. Ca gluconate and oral Ca carbonate were administered to all group I and II patients to maintain the proper serum Ca levels, but the 4 patients of group III received only Ca gluconate and Ca carbonate after PTX to avoid severe hypocalcemia. Serum Ca levels were measured once a couple of days after PTX. The dose of Ca administered was 57.5 ± 17.1 g (22.5 to 73.6 g) to the 15 group I patients and 37.0 ± 13.5 g (24.3 to 55.4 g) to group III patients during the first 4 weeks after PTX. Transiliac bone biopsy specimens were obtained from the left iliac crest before PTX and from the right iliac crest 3 weeks (*n* = 3) or 4 weeks (*n* = 12) after PTX in group I and before and 4 weeks after surgery in group III (*n* = 4). In addition, the 4 patients from group II (a subset of group I) underwent a third bone biopsy from the left iliac crest 1 year after PTX. As a result, the 4 group II patients received bone biopsies three times (presurgery, 3 to 4 weeks after PTX, and 1 year after PTX). The patients were included only after consent was obtained.

### Parathyroidectomy

Nineteen HD patients received PTX for renal hyperparathyroidism after failure or lack of vitamin D therapy, and four or five parathyroid glands were successfully removed under general anesthesia in the patients. After the surgery, 150 mg of diffuse hyperplastic parathyroid tissue were autotransplanted into the adipose tissue of the abdominal wall^7^, ^19^ to avoid complications caused by long‐term low bone turnover.^27^, ^28^, ^29^ In these 19 patients, serum intact PTH levels were measured by an immunoradiometric assay (Allegro Intact PTH; Nichols Institute Diagnostics, San Juan Capistrano, CA, USA) or an electrochemiluminescence immunoassay (Elecsys PTH; Roche Diagnostics, GmbH, Mannheim, Germany); intact PTH levels fell to 25 pg/mL or less 1 week after PTX. In 1 patient, three parathyroid glands were removed and serum intact PTH fell from 1092 to 212 pg/mL 1 week later and further to 76 pg/mL 4 weeks after PTX. In this patient, 50 mg of diffuse hyperplastic parathyroid tissue had been autotransplanted. Nodular hyperplastic parathyroid tissue was not autotransplanted as it is highly possible for recurrence of hyperparathyroidism to occur after PTX.^30^ All patients resumed walking 2 days after PTX: Mechanical loading is important to maintain bone properties.^31^, ^32^, ^33^ These patients were treated with PTX with immediate autotransplantation between 1996 and 2010.

### Serum bone metabolism parameters

The serum levels of intact PTH, tartrate‐resistant acid phosphatase (TRACP; means of colorimetry, using ρ‐nitrophenyl phosphate as the substrate), deoxypyridinoline (DPD; high‐performance liquid chromatography), carboxy‐terminal propeptide of human type I procollagen (PICP; radioimmunoassay kit, PICP ORION; Orion Diagnostica, Espoo, Finland), and total alkaline phosphatase (total‐ALP; means of colorimetry, using ρ‐nitrophenyl phosphate as the substrate) were measured before and 3.8 ± 0.4 (3 or 4) weeks after PTX in groups I and II, and before and 4 weeks after PTX in group III, although serum bone metabolism parameters are sometimes not reliable in HD patients.^34^ Serum‐intact PTH, Ca, and P levels were measured and compared at 4 weeks and 1 year after PTX in group II (Table 2). Blood samples were collected immediately before PTX and immediately before the iliac bone biopsies performed 4 weeks after PTX. Plasma 1.25(OH)_2_D_3_ (radioimmunoassay kit; Immuno Diagnostic Systems Ltd, Boldon, UK) levels were measured in group III patients, but not in groups I or II.

**Table 2:** Bone Metabolism Parameters Measured Before and 3.8 ± 0.4 (3 or 4) Weeks After PTX in Group I (*n* = 15)

	Pre‐PTX	Post‐PTX (3 or 4 weeks)	*p* Value	Power (%)	Normal values
Intact PTH (pg/mL)	1210.5 ± 461.2	18.1 ± 21.3	<0.001	100.0	10.0–65.0
TRACP (U/L)	23.4 ± 8.6	7.3 ± 3.0	<0.001	100.0	5.5–17.2
DPD (pmol/mL)	57.7 ± 65.7	9.3 ± 4.1	<0.001	90.9	Unknown
PICP (ng/mL)	380.3 ± 457.0	475.3 ± 367.4	0.039	54.1	30.0–182.0
Total ALP (U/L)	766.3 ± 576.8	950.5 ± 518.5	0.004	86.4	85.0–255.0
Ca (mg/dL)	9.9 ± 0.8	10.4 ± 1.8	NS	32.4	8.4–10.4
P (mg/dL)	5.5 ± 1.3	3.0 ± 1.6	0.002	96.5	2.5–4.5

PTX = Total parathyroidectomy; TRACP = tartrate‐resistant acid phosphatase; DPD = deoxypyridinoline; PICP = carboxy‐terminal propeptide of human type I procollagen; ALP = alkaline phosphatase; NS = nonsignificant.

### Bone histomorphometry

Tetracycline hydrochloride (Japan Lederle, Tokyo, Japan) was administered for 2 or 3 days with an interlabel period of 8 to 13 days. A 3‐ to 4‐day‐washout period was allowed before the biopsy was taken in the patients. Static and dynamic histomorphometric parameters defined by Dempster and colleagues^35^ and Recker and colleagues^36^ were measured in the central portion of cancellous bone. In addition, the hypomineralized bone area normalized to total bone area (hypomineralized bone area; HM.B.Ar/B.Ar; %)^10^, ^11^ was measured at the central portion of the cancellous bone by fluorescent light microscopy.

Osteoclast surface (Oc.S/BS), eroded surface (ES/BS), osteoblast surface (Ob.S/BS), and osteoid surface (OS/BS) were measured in cancellous bone and statistically compared before and after PTX in all groups, and also at 4 weeks and 1 year after PTX in group II. The bone formation rate normalized to the bone surface (BFR/BS; mm^3^/mm^2^/year) was measured 3 to 4 weeks after PTX in groups I and III. However, BFR/BS was not measured 1 year after PTX in group II. Hypomineralized bone area was measured in cancellous bone to compare the values obtained before and 3 to 4 weeks after PTX in all groups, and 4 weeks and 1 year after PTX in group II.

### Raman spectroscopy

Embedded biopsies were sanded to create a flat surface and then polished with a 3‐μm diamond suspension followed by a 50‐nm alumina suspension. We did Raman measurements from the same sections as those used form histology, but were not able to specifically target hypomineralized areas for measurement. Bone near edges was likely more plasticized than tissue in more centrally located regions, so all measures were targeted toward bone away from edges. However, given the current laser set‐up on the Raman scope, we were not able to visualize any labels. Bone samples from 9 patients were chosen for the Raman spectroscopy because these were the only patients for whom we could get informed consent to perform another analysis. Also, several patients had died before we were able to ask their permission for this analysis. Raman spectroscopy was performed using an InVia Raman Spectrometer (Renishaw, Wotton‐under‐Edge, UK). A 785‐nm laser was focused on the bone surface using a 50× objective to a spot size of approximately 1.3 μm. Eight sites within the trabecular regions were imaged approximately 1 mm apart. Spectra were acquired following a 12‐sec exposure and were averaged across 12 accumulations. Baseline correction was accomplished using Renishaw WiRE software intelligent fitting. Gaussian peaks were fit to the PO_4_^3^‐ν1 peak and CO_3_^2^‐ν1 peaks by second‐derivative spectroscopy in GRAMS/AI (Thermo‐Fisher Scientific, Waltham, MA, USA). The extent of matrix mineralization was determined by the mineral:matrix ratio, calculated as the integrated area ratio of PO_4_^3^‐ν1 : Amide I. Mineral maturity: crystallinity was calculated as the inverse of the full width at half height of the PO_4_^3^‐ν1 Gaussian peak. Type B carbonate substitution was calculated as the Gaussian peak area ratio of CO_3_^2^‐ν1 : PO_4_^3^‐ν1. The eight spectra acquired for each sample were averaged, yielding a single value for each Raman parameter to be used for analysis.^37^

Informed consent was obtained from all patients after they were provided with a detailed explanation of both the risks and possible outcomes of bone biopsies and PTX. The procedure was conducted in accordance with the Declaration of Helsinki. The Institutional Review Board of Towa Hospital and its affiliated hospitals approved the study protocol.

### Statistical analysis

Statistical analyses were conducted using JMP 13 (SAS Institute Inc., Cary, NC, USA). The parameters pertaining to serum bone metabolism, histomorphometric parameters of bone turnover and hypomineralized bone area, and parameters obtained by Raman spectroscopy were compared with each other using nonparametric tests (Mann–Whitney tests) or unpaired Student's *t* tests. The parameters obtained by Raman spectroscopy were also compared before and after PTX using the Mann–Whitney test. Power analyses were computed with JMP (SAS Institute) post hoc using calculated means, standard deviations, and sample size for each group and variable, and presented in Tables 2, 3, 4, 5, 6, 7, 8, 9, 10.

**Table 3:** Bone Metabolism Parameters Measured 4 Weeks and 1 Year After PTX in Group II (*n* = 4)

	Post‐PTX (4 weeks)	Post‐PTX (1 year)	*p* Value	Power (%)	Normal values
Intact PTH (pg/mL)	6.8 ± 3.5	20.5 ± 15.0	NS	32.9	10.0–65.0
Ca (mg/dL)	12.3 ± 1.5	8.8 ± 0.5	NS	10	8.4–10.4
P (mg/dL)	2.3 ± 1.2	4.2 ± 1.7	NS	46.1	2.5–4.5

PTX = Total parathyroidectomy.

**Table 4:** Bone Metabolism Parameters Measured Before and 4 Weeks After PTX in Group III (*n* = 4)

	Pre‐PTX	Post‐PTX (4 weeks)	*p* Value	Power (%)	Normal values
Intact PTH (pg/mL)	961.5 ± 288.2	24.5 ± 15.7	0.125	98.5	10.0–65.0
TRACP (U/L)	24.2 ± 9.2	9.9 ± 1.9	0.125	66.8	5.5–17.2
DPD (pmol/mL)	26.8 ± 7.1	6.3 ± 1.4	0.125	73	Unknown
PICP (ng/mL)	300.3 ± 69.8	378.0 ± 104.0	0.125	30.2	30.0–182.0
Total ALP (U/L)	394.8 ± 106.6	461.8 ± 123.4	0.375	10.2	85.0–255.0
Ca (mg/dL)	9.9 ± 0.4	9.6 ± 1.1	0.5	8.4	8.4–10.4
P (mg/dL)	5.7 ± 0.8	2.8 ± 0.6	0.125	98.6	2.5–4.5
1.25 (OH)_2_D_3_ (pg/mL)	(−)	5.3 ± 2.2	(−)	(−)	20.0–60.0

Plasma 1.25 (OH)_2_D_3_ levels were measured 1 week after PTX in group III.

PTX = Total parathyroidectomy; TRACP = tartrate‐resistant acid phosphatase; DPD = deoxypyridinoline; PICP = carboxy‐terminal propeptide of human type I procollagen; ALP = alkaline phosphatase.

**Table 5:** Histomorphometric Parameters on Bone Turnover and Hypomineralized Bone Area Before and 3.8 ± 0.4 (3 or 4) Weeks After PTX in Group I (*n* = 15)

	Pre‐PTX	Post‐PTX (3 or 4 weeks)	*p* value	Power (%)	Normal values
Oc.S/BS (%)	4.7 ± 3.7	0.2 ± 0.5	< 0.001	99.2	0.7 ± 0.7 (0.0–2.0)
ES/BS (%)	26.8 ± 12.0	3.2 ± 2.8	<0.001	100	4.0 ± 2.0 (1.75–7.00)
Ob.S/BS (%)	23.6 ± 11.7	17.9 ± 21.4	0.083	44.2	4.4 ± 3.2 (0.0–9.5)
OS/BS (%)	49.2 ± 17.1	78.8 ± 25.3	0.003	95.5	14.3 ± 6.3 (7.0–25.0)
BFR/BS (mm^3^/mm^2^/year)	(−)	0.020 ± 0.015	(−)	(−)	0.014 ± 0.008 (0.001–0.016)
HM.B.Ar/B.Ar (%)	17.3 ± 12.8	2.6 ± 3.2	<0.001	99.6	(−)

PTX = Total parathyroidectomy; Oc.S/BS = osteoclast surface; ES/BS = eroded surface; Ob.S/BS = osteoblast surface; OS/BS = osteoid surface; BFR/BS = bone formation rate normalized to bone surface; HM.B.Ar/B.Ar = hypomineralized bone area.

**Table 6:** Histomorphometric Parameters on Bone Turnover and Hypomineralized Bone Area 4 Weeks and 1 Year After PTX in Group II (*n* = 4)

	Post‐PTX (4 weeks)	Post‐PTX (1 year)	*p* Value	Power (%)	Normal values
Oc.S/BS (%)	0	0.1 ± 0.1	0.182	28.9	0.7 ± 0.7 (0.0–2.0)
ES/BS (%)	2.3 ± 1.8	7.7 ± 6.1	0.25	75.5	4.0 ± 2.0 (1.75–7.00)
Ob.S/BS (%)	3.1 ± 2.4	0	0.25	42.9	4.4 ± 3.2 (0–9.5)
OS/BS (%)	73.9 ± 15.5	58.3 ± 31.9	0.625	11.1	14.3 ± 6.3 (7.0–25.0)
HM.B.Ar/B.Ar (%)	2.5 ± 1.8	14.4 ± 5.0	0.125	84.8	(−)

PTX = total parathyroidectomy; Oc.S/BS = osteoclast surface; ES/BS = eroded surface; Ob.S/BS = osteoblast surface; OS/BS = osteoid surface; BFR/BS = bone formation rate normalized to bone surface; HM.B.Ar/B.Ar = hypomineralized bone area.

**Table 7:** Histomorphometric Parameters on Bone Turnover and Hypomineralized Bone Area Before and 4 Weeks After PTX in Group III (*n* = 4)

	Pre‐PTX	Post‐PTX (4 weeks)	*p* Value	Power (%)	Normal values
Oc.S/BS (%)	7.4 ± 3.4	0.2 ± 0.3	0.125	75.5	0.7 ± 0.7 (0.0–2.0)
ES/BS (%)	30.2 ± 9.8	2.2 ± 3.9	0.125	84.5	4.0 ± 2.0 (1.75–7.00)
Ob.S/BS (%)	31.9 ± 8.2	26.9 ± 11.7	0.625	9.2	4.4 ± 3.2 (0.0–9.5)
OS/BS (%)	54.1 ± 10.1	81.9 ± 18.0	0.125	36.1	14.3 ± 6.3 (7.0–25.0)
BFR/BS (mm^3^/mm^2^/year)	(−)	0.026 ± 0.033	(−)	(−)	0.014 ± 0.008 (0.001–0.016)
HM.B.Ar/B.Ar (%)	6.0 ± 4.0	14.3 ± 3.5	0.125	49.5	(−)

Normal values were referenced from ref 36.

PTX = total parathyroidectomy; Oc.S/BS = osteoclast surface; ES/BS = eroded surface; Ob.S/BS = osteoblast surface; OS/BS = osteoid surface; BFR/BS = bone formation rate normalized to bone surface; HM.B.Ar/B.Ar = hypomineralized bone area.

**Table 8:** Relationship Between Histomorphometric Parameters of Bone Turnover and Hypomineralized Bone Area Obtained After PTX in Group I (*n* = 15) and Those Obtained After PTX in Group III (*n* = 4)

	Group I	Group III	*p* Value	Power (%)	Normal values
Oc.S/BS (%)	0.2 ± 0.5	0.2 ± 0.3	0.393	(−)	0.7 ± 0.7 (0.0–2.0)
ES/BS (%)	3.2 ± 2.8	2.2 ± 3.9	0.21	95.4	4.0 ± 2.0 (1.75–7.00)
Ob.S/BS (%)	17.9 ± 21.4	26.9 ± 11.7	0.024	8.5	4.4 ± 3.2 (0.0–9.5)
OS/BS (%)	78.8 ± 25.3	81.9 ± 18.0	1	5.3	14.3 ± 6.3 (7.0–25.0)
BFR/BS (mm^3^/mm^2^/year)	0.020 ± 0.015	0.026 ± 0.033	0.952	5.6	0.014 ± 0.008 (0.001–0.016)
HM.B.Ar/B.Ar (%)	2.6 ± 3.2	14.3 ± 3.5	0.004	98.9	(−)

Normal values were referenced from ref 36.

PTX = total parathyroidectomy; Oc.S/BS = osteoclast surface; ES/BS = eroded surface; Ob.S/BS = osteoblast surface; OS/BS = osteoid surface; BFR/BS = bone formation rate normalized to bone surface; HM.B.Ar/B.Ar = hypomineralized bone area.

**Table 9:** Relationship Between Parameters on Raman Spectroscopy Obtained Before and After PTX in Group I (*n* = 6)

	Pre‐PTX	Post‐PTX	*p* Value	Power (%)
PO_4_^3^‐ν1 : Amide I	2.317 ± 0.346	2.346 ± 0.333	NS	6.0
CO_3_^2^‐ν1 : PO_4_^3^‐ν1	0.222 ± 0.019	0.232 ± 0.027	NS	17.1
Crystallinity : maturity	0.063 ± 0.002	0.062 ± 0.002	NS	50.7

PTX = total parathyroidectomy; PO_4_^3^‐ν1 : Amide I = mineral:matrix ratio; CO_3_^2^‐ν1 : PO_4_^3^‐ν1 = type B carbonate substitution; NS = nonsignificant.

**Table 10:** Relationship between Parameters on Raman Spectroscopy Obtained Before and After PTX in Group III (*n* = 3)

	Pre‐PTX	Post‐PTX	*p* Value	Power (%)
PO_4_^3^‐ν1 : Amide I	2.266 ± 0.172	2.646 ± 0.288	NS	25.0
CO_3_^2^‐ν1 : PO_4_^3^‐ν1	0.255 ± 0.019	0.222 ± 0.034	NS	16.6
Crystallinity : maturity	0.062 ± 0.001	0.061 ± 0.002	NS	8.4

PTX = total parathyroidectomy, PO_4_^3^‐ν1 : Amide I = mineral:matrix ratio; CO_3_^2^‐ν1 : PO_4_^3^‐ν1 = type B carbonate substitution; NS = nonsignificant.

## Results

### Serum bone metabolism parameters

Serum resorption parameters, including TRACP and DPD levels decreased and serum formation parameters, including PICP and total‐ALP levels significantly increased after PTX in group I (Table 2). Serum resorption parameters decreased and serum formation parameters increased in the 4 patients in group III (Table 4), but neither of these differences was significant. Serum Ca levels remained unchanged, probably because they were maintained by i.v. Ca gluconate and oral Ca carbonate administration after PTX. Serum P levels significantly decreased after PTX in both groups I and III (Tables 2 and 4). Plasma 1.25(OH)_2_D_3_ levels were extremely low in group III patients [5.3 ± 2.2 (2.8 to 7.9) pg/mL]; the normal range of plasma 1.25(OH)_2_D_3_ levels is from 20 to 60 pg/mL.

### Histomorphometry of bone turnover

Resorption parameters decreased after PTX in group I (Table 5). Although Ob.S/BS was not significantly different before and after PTX (*p* = 0.083), OS/BS increased in group I, possibly because of hypophosphatemia and a failure to mineralize newly deposited matrix (Table 5). In group II, Oc.S/BS increased from 0 to 0.1% 1 year after PTX (Table 6.), probably because serum intact PTH increased by threefold 1 year after surgery (Table 3). Bone resorption parameters (Oc.S/BS and ES/BS) decreased in all 4 patients after PTX in group III, although the changes were not statistically significant (Table 7.), probably because of the small sample size. BFR/BS was 0.020 ± 0.015 (0.003 to 0.032) mm^3^/mm^2^/year in group I (Table 5) and was 0.026 ± 0.033 (0 to 0.070) mm^3^/mm^2^/year in group III (Table 7.) 4 weeks after PTX, but there was no statistical difference between these two groups even though Ob.S/BS was significantly greater in group III than in group I after PTX (Table 8.).

### Histomorphometry of hypomineralized bone area

**Figure 2:**
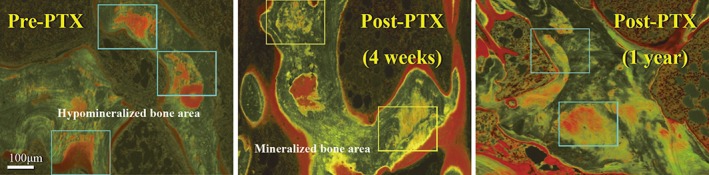
Hypomineralized bone area before and after total parathyroidectomy (PTX) in group I. Hypomineralized bone area was observed in a hemodialysis patient suffering from renal hyperparathyroidism (*A*). Reduction of hypomineralized bone area was observed 4 weeks after PTX with immediate autotransplantation of the diffuse hyperplastic parathyroid tissue followed by 2.0 μg/day of alfacalcidol administration in a group I patient. Mineralization within bone matrix presumably mediated by the osteocytic perilacunar/canalicular system was observed 4 weeks after PTX. Mineralization within the matrix was activated by alfacalcidol administration (central photo). The single labelings caused by osteoblasts are also shown (*B*). Thereafter, hypomineralized bone area was increased again 1 year after PTX after reduction of alfacalcidol from 2.0 to 0.5 μg/day (*C*; group II). The lower dose of alfacalcidol is insufficient to maintain normal mineralization by osteocytes.

**Figure 3:**
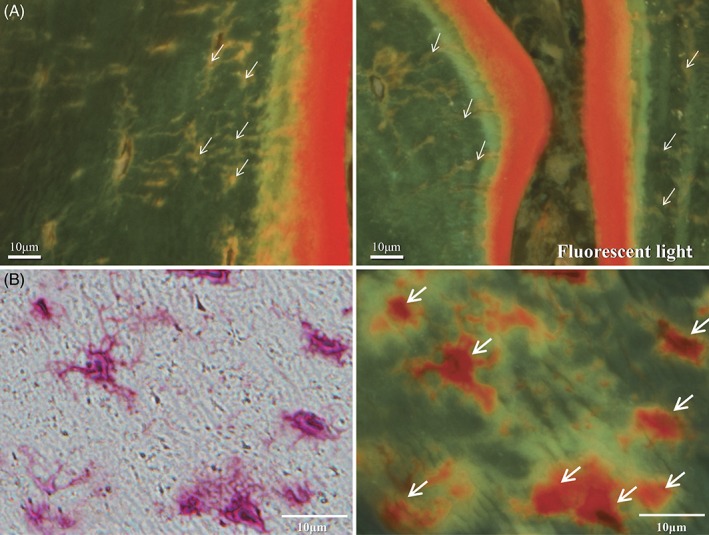
(*A*) Osteocyte canaliculi after total parathyroidectomy (PTX) in group I. The osteocyte canaliculi stained by tetracycline hydrochloride were clearly visible in a group I patient after PTX followed by 2.0 μg/day of alfacalcidol administration. It is likely that mineralization within bone matrix presumably mediated by the osteocytic perilacunar/canalicular system was maintained if the hemodialysis patients receive 2.0 μg/day of alfacalcidol after PTX. White arrows are pointing to osteocyte canaliculi stained by tetracycline hydrochloride. (*B*) Osteocyte canaliculi after PTX in group I. Osteocyte lacunae are shown and those on the right are stained by tetracycline hydrochloride. White arrows are pointing to osteocyte lacunar walls stained by tetracycline hydrochloride.

**Figure 4:**
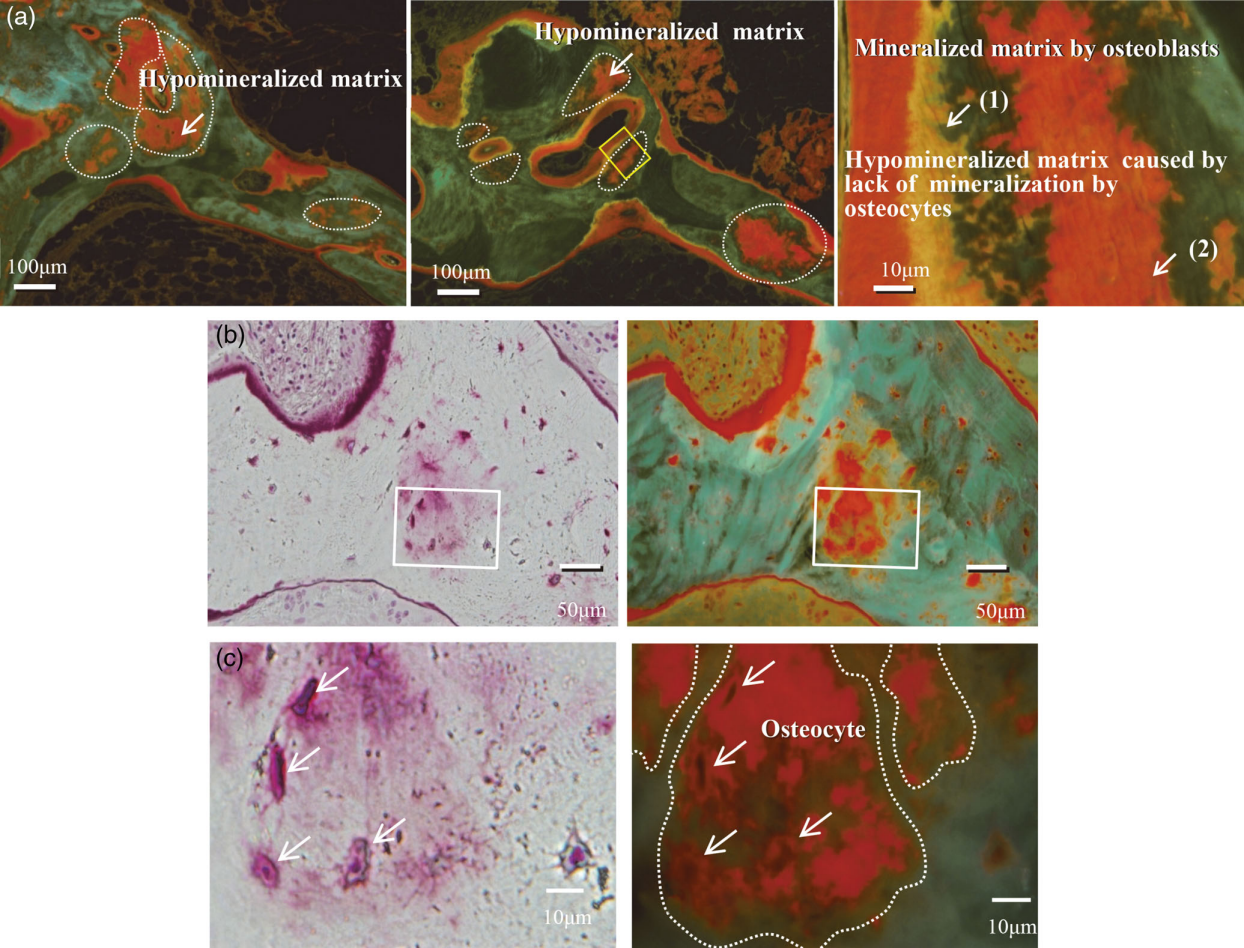
(*A*) Hypomineralized bone area before and after PTX in group III (upper panels). Increased hypomineralized bone area 4 weeks after PTX was found in a group III patient not receiving alfacalcidol administration. The schema on the right shows both the single labelings caused by osteoblasts and hypomineralized bone area caused by osteocytes. These results indicate that vitamin D is required at least in moderate doses for adequate mineralization by osteocytes and osteoblasts. Mineralization in the osteocytic perilacunar/canalicular system was not observed around the osteocyte lacunae in a patient not receiving alfacalcidol. White arrows are pointing to (1) mineralized matrix by osteoblasts, and (2) hypomineralized matrix caused by lack of mineralization by osteocytes. (*B*, *C*) Higher‐resolution images of osteocyte lacunae. Higher‐resolution images of osteocyte lacunae within the hypomineralized bone area, as well as osteoblasts on top of the surface of the respective area, are shown. White arrows are pointing to hypomineralized matrix area.

HM.B.Ar/B.Ar significantly decreased from 17.3 ± 12.8 to 2.6 ± 3.2% (*p* = 0.001) after PTX in the group I patients (Table 5, Figs. 2, 3 *A*, and 3*B*). These parameters were also compared between 4 weeks and 1 year after PTX in the 4 patients who underwent a third biopsy (group II) after the dose of alfacalcidol was reduced from 2.0 to 0.5 μg/day at 4 weeks. HM.B.Ar/B.Ar increased in all 4 patients (2.5 ± 1.8 versus 14.4 ± 5.0%, *p* = 0.125) 1 year after PTX (Table 6., Fig. 2). In group III, who did not receive alfacalcidol after PTX, HM.B.Ar/B.Ar increased in all 4 patients (6.0 ± 4.0 versus 14.3 ± 3.5%, *p* = 0.125) (Table 7., Fig. 4 *A* to 4*C*). HM.B.Ar/B.Ar was significantly greater in group I than in group III after PTX (Table 8).

### Raman spectroscopy for mineral:matrix ratio, mineral maturity, and crystallinity

**Figure 5:**
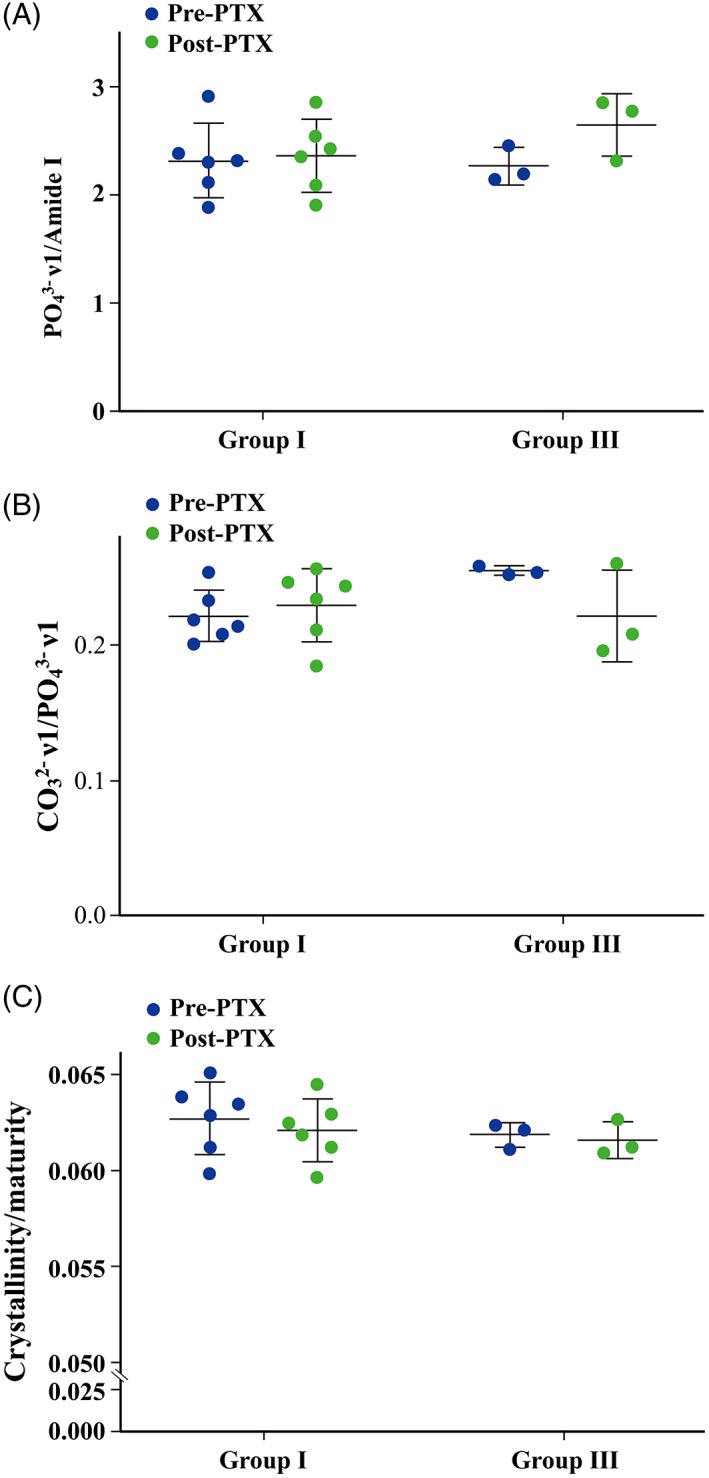
(*A*) PO_4_^3^‐ν1 : Amide I values before and after PTX in groups I and III. Mineral:matrix ratio is evaluated and shown as PO_4_^3^‐ν1 : Amide I. The mineral:matrix ratio did not change following PTX surgery in group I. However, although the mineral:matrix ratio increased in all 3 patients after surgery in group III, the change in this parameter was not significant probably because of the limitation of statistical power in this group (Tables 9. and 10). (*B*) CO_3_^2^‐ν1 : PO_4_^3^‐ν1 values before and after PTX in groups I and III. The maturity of mineral deposited to matrix, defined by its carbonate content, was evaluated and is presented as CO_3_^2^‐ν1 : PO_4_^3^‐ν1. Mineral maturity did not change following PTX surgery in group I. Although mineral maturity decreased from 0.259 to 0.196 and 0.254 to 0.209 in 2 of 3 patients after PTX in group III, the difference was not significant. (*C*) Crystallinity/maturity values before and after PTX in groups I and III. Crystallinity did not change after PTX in either group I or group III. However, crystallinity is not expected to change within 3 to 4 weeks after PTX.

The mineral:matrix ratio, shown as PO_4_^3^‐ν1 : Amide I, did not change following PTX surgery in group I (*n* = 6; 2.317 ± 0.346 before PTX and 2.346 ± 0.333 after surgery) (Fig. 5 *A* and Table 9.). Although the mineral:matrix ratio increased in all 3 patients after surgery in group III (2.266 ± 0.172 versus 2.646 ± 0.288) (Fig. 5 *A*), the change in this parameter was not significant (Table 10). Mineral maturity, defined by the bone's carbonate content and shown as CO_3_^2^‐ν1 : PO_4_^3^‐ν1, did not change following PTX surgery in group I (*n* = 6; 0.222 ± 0.019 before PTX and 0.232 ± 0.027 after surgery) (Fig. 5 *B*, Table 9). Although mineral maturity decreased from 0.259 to 0.196 and 0.254 to 0.209 in 2 of 3 patients in group III, the difference was not significant (0.255 ± 0.019 before PTX versus 0.222 ± 0.034 after surgery) (Fig. 5 *B*, Table 10). Crystallinity of the mineral is shown in Fig. 5 *C*. Crystallinity did not change post‐PTX surgery in either group I or group III (Fig. 5 *C*, Tables 9. and 10). However, crystallinity is not expected to change within 3 to 4 weeks after PTX.

## Discussion

Development of low‐turnover osteomalacia following PTX is generally observed in patients with renal hyperparathyroidism.^7^, ^19^, ^29^, ^34^ We previously demonstrated that mineralization around the osteocyte cell bodies and canaliculi was greater than that at the mineralization front after PTX followed by 2.0 μg/day of alfacalcidol administration.^7^ This finding is highly relevant and important as one of the causes of hypocalcemia and hypophosphatemia, namely, hungry bone syndrome after PTX in group I.^7^

Matrix mineralization by osteocytes may be disturbed by various factors, including vitamin D deficiency^11^ and hypophosphatemia.^38^ In this study of patients with renal hyperparathyroidism, hypomineralized matrix was found in large bone areas probably because of a severe mineralization deficiency (Fig. 2 *A*).^10^, ^11^ Following PTX, the hypomineralized bone matrix around osteocytes was reduced within 4 weeks by administration of 2.0 μg/day of alfacalcidol administration. However, when the dose was reduced to 0.5 μg/day of alfacalcidol 4 weeks after PTX to avoid long‐term consequences of low‐turnover bone disease with hypoparathyroidism, the area of hypomineralized bone increased by sixfold over the course of the following year (group II). When no alfacalcidol was administered after PTX (group III), the area of hypomineralized bone doubled within 4 weeks following PTX. The higher dose of biologically active vitamin D clearly suppresses the function of parathyroid tissue,^12^, ^13^ but the lower dose of vitamin D or absence of vitamin D after PTX leads to increased hypomineralized bone area (Fig. 4 *C*). It is also possible that osteoid formation on the osteocyte lacunar walls continued after PTX, contributing to the increased hypomineralized bone matrix. The vitamin D receptor is expressed on osteocytes,^21^ and biologically active vitamin D may stimulate the receptor on the osteocyte, leading to increased matrix mineralization.

These results indicate that vitamin D is required at least in moderate doses for adequate mineralization by osteocytes (Tables 5. to 8, Figs. 2, 3, 4). As a future study, whether the vitamin D sterols, including paricalcitol (19‐nor‐1α.25‐dihydroxyvitamin D_2_; AbbVie Inc. North Chicago, IL, USA), activate mineralization by osteocytes should be investigated.

Raman spectroscopy was used to further characterize changes in the perilacunar matrix around osteocytes. Raman spectroscopy showed that mineralized matrix did not change significantly in patients whether or not they received alfacalcidol after PTX. Crystallinity of the deposited mineral did not change either, but this was an expected result only 3 to 4 weeks after PTX. This analysis showed that mineralized matrix did not change significantly in patients whether or not they received alfacalcidol after PTX. Crystallinity of the deposited mineral did not change either, but this was an expected result only 3 to 4 weeks after PTX.

The biopsies used here were embedded in plastic prior to Raman imaging. When sanding and polishing a surface for Raman, the plastic surface is flattened, which then exposes some of the bone's surface. In practice, patches of bone were exposed on a given surface, and even a small amount of surface plastic overlaying a section of bone will distort the Raman spectrum. In addition, given that the bone was embedded, bone near any surface, including near any osteocyte lacunae, would be plasticized, which can alter this type of characterization.

The second limitation of the study is that the postyear 1 bone biopsy was performed only in group II patients. The additional limitation of the analysis is that the subsample was small, and we could not get permission for all of the patients in the study to perform this analysis.

Based on these results, we suggest that that PTX followed by 2.0 μg/day of alfacalcidol administration is an important option to increase BMD in HD patients with hyperparathyroid bone disease caused by renal hyperparathyroidism. Biologically active vitamin D may be required at least in moderate doses for adequate mineralization by osteocytes to reduce hypomineralized bone area. However, a larger study is needed to demonstrate this conclusively. Further study of osteocyte‐specific histological parameters and perilacunar changes in response to PTX and vitamin D administration in patients with renal hyperparathyroidism could further elucidate the role that osteocytes play in regulating bone matrix mineralization.

## Disclosures

The authors state that they have no conflicts of interest.
